# Draft Genome Sequence of *Lactobacillus plantarum* WLPL04, Isolated from Human Breast Milk

**DOI:** 10.1128/genomeA.01443-15

**Published:** 2015-12-10

**Authors:** XueYing Tao, Meiling Jiang, Feng Zhang, Feng Xu, Hua Wei

**Affiliations:** a State Key Laboratory of Food Science and Technology, Nanchang University, Nanchang, China; b School of Life Sciences, Nanchang University, Nanchang, China

## Abstract

*Lactobacillus plantarum* WLPL04, a novel strain, was isolated from a breast milk sample from a healthy woman and demonstrated several probiotic functions. Here, we present the draft genome sequence of this strain, which contains 3,192,587 bp, a G+C content of 44.52%, 3,158 protein-coding genes, and 53 tRNA genes.

## GENOME ANNOUNCEMENT

*Lactobacillus plantarum* is a Gram-positive lactic acid bacterium commonly found in various ecological niches, e.g., pickles, sausage, sourdough, wine, and the gastrointestinal tract; it is considered to be an important probiotic species, since some strains of *L. plantarum* possess a variety of health benefits (1–4). Here, we report the draft genome of *L. plantarum* strain WLPL04, isolated from the breast milk of a healthy woman. This strain was found to possess survival capacities (acid and bile salt tolerance and survival in simulated gastrointestinal tract) and probiotic properties (inhibition of pathogens, antiadhesion of pathogens, and protection from SDS harmful effects and inflammatory stress on Caco-2 cells).

Whole-genome sequencing of *L. plantarum* WLPL04 was performed by Shanghai Majorbio Bio-pharm Technology Co., Ltd. (Shanghai, China) using an Illumina HiSeq 4000 with 300-bp paired-end reads. The reads were trimmed and assembled *de novo* using SOAP*denovo* 2.04 (http://soap.genomics.org.cn/). The assembly yielded 301-fold coverage of a 3,192,587-bp draft genome contained in 72 contigs and 67 scaffolds, with an average G+C content of 44.52%. The contig *N*_50_ was approximately 190.7 kb, and the largest contig assembled was approximately 606.6 kb.

The prediction of open reading frames (ORFs) was performed by using Glimmer version 3.02 (http://www.cbcb.umd.edu/software/glimmer/), and gene annotation was carried out using the Prokaryotic Genomes Automatic Annotation Pipeline (PGAAP) and BLASTP (5) provided by National Center for Biotechnology Information (NCBI). The genome of *L. plantarum* WLPL04 contains 3,158 candidate protein-coding genes (average size, 840 bp), giving a coding intensity of 83.1%. Predicted copies of the 16S and 23S rRNA genes and 53 genes for tRNAs were found. The sequence of the 16S rRNA gene (1,555 bp) of *L. plantarum* WLPL04 is 100% identical to that of *L. plantarum* subsp. *plantarum* ATCC 14917 (NCBI accession no. ACGZ01000098) and *L. plantarum* strain LP3 (NCBI accession no. AY675256.1). Among all the predicted proteins, 1,750 (80.5%) proteins were annotated with known functional Clusters of Orthologous Groups (COG) (6) categories, and 208 (9.6%) proteins were annotated as general function prediction only.

The draft genome sequence described in this report will help understand both how *L. plantarum* adapts to different environments and the mechanism of its probiotic properties.

### Nucleotide sequence accession numbers.

This whole-genome shotgun project has been deposited at DDBJ/EMBL/GenBank under the accession no. LKCO00000000. The version described in this paper is version LKCO01000000.
